# Sense of Coherence and Its Association With Adherence to Continuous Positive Airway Pressure (CPAP) Therapy in Patients With Obstructive Sleep Apnoea

**DOI:** 10.7759/cureus.115745

**Published:** 2026-09-03

**Authors:** Monserrat Gómez Juárez, Fátima K Gaytán Núñez, Francisco Vargas Hernandez, Maria G Saucedo Martínez, Moisés Moreno Noguez, Mauricio Rojas Ibañez, Kevin G Cortes Gonzalez, Jehu A Tamayo Calderon, Eva L Granados Franco, Everardo Villar Aguirre

**Affiliations:** 1 Family Medicine, Family Medicine Unit No. 64 "Tequexquinahuac" Instituto Mexicano del Seguro Social, Tlalnepantla de Baz, MEX; 2 Auxiliary Medical Coordination for Health Research, Eastern Mexico Regional Operational Administrative Body, Instituto Mexicano del Seguro Social, Tlalnepantla de Baz, MEX

**Keywords:** chronic respiratory diseases, cpap adherence, obstructive sleep apnoea (osa), obstructive sleep apnoea syndrome, sense of coherence

## Abstract

Introduction: Obstructive sleep apnoea (OSA) has a negative impact on cardiovascular and cognitive health. Although continuous positive airway pressure (CPAP) is the treatment of choice, non-adherence limits its effectiveness. Sense of coherence (SOC) is a salutogenic construct that facilitates the management of chronic conditions, although its specific relationship with the use of CPAP has been little studied.

Main objective: To determine the association between SOC and adherence to CPAP treatment in patients with OSA.

Materials and methods: A cross-sectional analytical study was conducted between February 2025 and July 2025 at a family medicine unit of the Mexican Social Security Institute (IMSS). The study included 200 subjects diagnosed with OSA. SOC was assessed using the SOC-29 questionnaire, and treatment adherence was assessed by reading the SD cards of the devices, defined as use for more than four hours per night on at least five days per week (70% of nights over 30 days). Multiple binary logistic regression was performed for treatment adherence, with Bootstrap resampling (2,000 samples), and the odds ratio (OR) and 95% confidence interval (CI) were calculated.

Results: One was found mean age was 62 (SD = 12) years, 60% (n = 120) were male, 36% (n = 72) had a primary education level, and 60% (n = 120) were married. A high SOC was identified in 74% (n = 148) of the sample, which was significantly associated with CPAP adherence (p < 0.001, Cramér's V = 0.473). In terms of weekly CPAP usage of fewer than five days, an OR of 8.538 (95% CI: 4.663-15.633) for low SOC was reported. In the multivariate model for non-adherence, low SOC remained the strongest independent risk factor for non-adherence (OR = 9.043, 95% CI: 4.359-18.761, p = 0.005).

Conclusion: The findings suggest that low SOC is a risk factor for non-adherence to treatment. It is a key clinical predictor for developing motivational strategies to improve treatment persistence in patients with OSA.

## Introduction

Obstructive sleep apnoea (OSA) is a disorder characterised by repeated episodes of partial or complete collapse of the upper airway during sleep, resulting in hypoxaemia, micro-awakenings, and fragmented sleep. Regarded as a global public health problem, it affects more than 936 million adults worldwide and is estimated to affect 27% of the population in Mexico [1,2]; it is more common in obese or elderly individuals. If OSA is not treated properly, it increases the risk of cardiovascular complications, metabolic changes, neurocognitive decline, and road traffic accidents due to daytime sleepiness [3].

The treatment of choice is continuous positive airway pressure (CPAP), and its effectiveness depends solely on adherence to at least four hours of use per night [4]; however, the dropout rate is around 50% [5]. Against this backdrop, Aaron Antonovsky's salutogenic theory proposes the sense of coherence (SOC) as a construct that explains an individual's ability to perceive their life as comprehensible, manageable, and meaningful [6]. A high SOC is a strong predictor of resilience and empowerment, which facilitates patients' management of their condition and reliable health behaviours, such as treatment adherence. Clinically, SOC assessment enables the early identification of individuals at risk of dropping out of treatment, allowing for the implementation of motivational strategies that reduce healthcare costs and social harm [7].

The primary component of the salutogenic model is the SOC, which is a global life view and a flexible disposition that reflects a person's strong and stable conviction that the internal and external environment is understandable and organized, and that events should unfold to meet one's expectations. SOC is not a fixed characteristic of a person's personality; it is a flexible manner in which individuals perceive the world and react to its pressures - for example, how they respond to the pressure to increase their adherence to CPAP therapy [8].

Despite its importance, there remains a gap in the international evidence linking SOC and CPAP adherence to objective adherence data obtained from devices. Therefore, the aim of this study was to investigate the association between SOC and CPAP adherence in people with sleep apnoea. A hypothesis of a moderate association between the factors is proposed.

## Materials and methods

Study design and participants

An analytical cross-sectional study was conducted at a family medicine unit, performing an initial screening of a database of 1,500 adult individuals diagnosed with OSA [3] who had been receiving CPAP therapy for at least three months between February 2025 and July 2025. After excluding records lacking documented diagnoses and those without clinical follow-up, 634 patients remained. Subsequently, exclusion criteria were applied, resulting in a total of 200 participants included in the analysis, from outpatients attending Family Medicine Unit No. 64 Tequesquinahuac, Instituto Mexicano del Seguro Social (IMSS), México.

**Figure 1:**
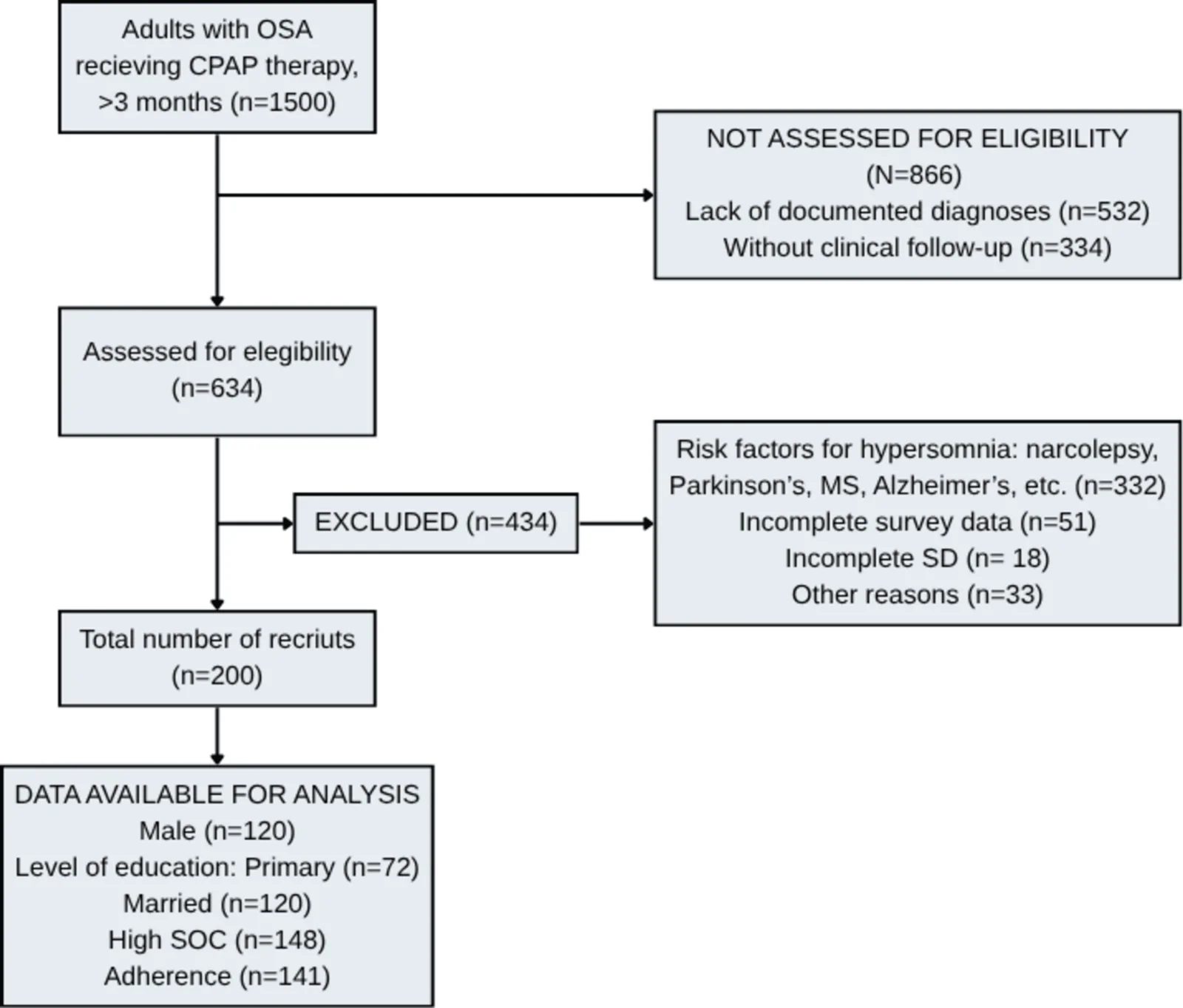
Flowchart of OSA patients in the Family Medicine Unit No. 64 "Tequesquinahuac" STROBE: Strengthening the Reporting of Observational Studies in Epidemiology The figure was created by the authors using Canva (version 2026; Canva Pty Ltd., Sydney, Australia).

Inclusion criteria consisted of adult patients diagnosed with OSA who had been receiving CPAP treatment for at least three months. Subjects with risk factors for hypersomnia, including medical conditions (narcolepsy, Parkinson's disease, multiple sclerosis, Alzheimer's disease), were excluded, and those with incomplete information were eliminated (Figure 1).

SOC was assessed using the SOC-29 questionnaire [8]. This questionnaire evaluates Antonovsky's salutogenic components (comprehensibility, manageability, and meaningfulness) on a 7-point Likert scale, with total scores ranging from 29 to 203 points [8]. Higher scores indicate a stronger SOC. Since no universal cutoff point exists for this measure, the categories and cutoffs in our study population were defined using the estimated tertiles of the sample: low SOC: <108 points, moderate SOC: 109-124 points, and high SOC: >125 points. In the bivariate analyses, SOC was analyzed using the three-category classification. Adherence was assessed by reading the SD cards from the devices, defined as use for more than four hours per night on at least 70% of days [9]. 

Data collection

Information was obtained from primary sources by administering standardised instruments to subjects with OSA attending the family medicine outpatient clinic at Family Medicine Unit No. 64. Sociodemographic, clinical, and data relating to treatment adherence and SOC were collected.

The sample size was calculated using the formula for the difference in proportions regarding the expected outcome (non-adherence), assuming a 95% confidence level and 80% power (1-β) with a 1:1 ratio, and considering an expected outcome prevalence of 40% among exposed individuals (high SOC) and 60% among unexposed subjects (low SOC), similar to what was reported by Huang et al., 63.1% medication adherence and 36.9% medication non-adherence patients with T2D [10]. In addition, the sample was selected using non-probabilistic convenience sampling.

Data analysis was carried out using the statistical software Statistical Product and Service Solutions (SPSS, version 27; IBM SPSS Statistics for Windows, Armonk, NY), employing descriptive statistics and tests of association, with a pre-established level of significance. 

Definitions of variables

Treatment adherence and SOC were evaluated in patients with OSA using validated instruments. Adherence was quantified as hours of CPAP usage, with adequate adherence defined as device utilization exceeding four hours per day on at least five days per week (corresponding to 70% of nights over a 30-day evaluation period) [9].

The information was confirmed through the use of standardised questionnaires, ensuring the validity of the data collected.

Statistical analysis

SPSS was used. For the analysis of qualitative variables (SOC, treatment adherence, gender, educational level, socioeconomic status, marital status), frequency and percentage measures were obtained. Additionally, the quantitative variables used were the following: age, apnoea-hypopnoea index (AHI), hours of night-time use, days of use per week, and use per month.

To associate the variables with treatment adherence (adherence/non-adherence) with the SOC (strong, moderate, low), an analysis was carried out for the outcome of CPAP adherence, yielding p-values, odds ratios (OR), and Cramér's V. For the bivariable binary logistic regression, SOC was dichotomized into low SOC versus high SOC (grouping moderate and high levels as a reference). This was done to specifically evaluate low SOC as a risk factor for non-adherence to treatment, as well as to maintain adequate statistical significance and stability during bootstrapping.

A multiple binary logistic regression model was constructed using bootstrapping (2,000 resamples) for the outcome of CPAP non-adherence. The following were considered as covariates: high SOC, single marital status, age under 65 years old, basic educational level, and male gender. ORs were calculated, along with 95% CIs and p-values.

Data imputation

A simple imputation method was used. For quantitative variables with a normal distribution, the mode was used. For qualitative variables, the most frequent category was used. Only data points with missing values below 20% were imputed. No data were imputed for the "sense of coherence" or "adherence" variables.

Ethical considerations

This study was reviewed and approved by the Local Health Research Committee 1408 and the corresponding Research Ethics Committee on January 30, 2025 (Registration No. R-2025-1408-002). The study complied with all ethical mandates, requiring written informed consent from each participating patient prior to enrollment. The authors declare no conflicts of interest.

## Results

Of a total of 200 participants, the average age was 62 years (12%). An average usage time of seven hours was found, with a residual AHI of 0.84; 60% (n = 120) were male. Regarding educational level and marital status, 36% (n = 72) reported having completed primary education, and 60% (n = 120) stated that they were married. A high SOC was observed in 74% (n = 148) of participants, and treatment adherence in 70.5% (n = 141) (Table 1).

**Table 1:** Sociodemographic characteristics of subjects with sleep apnoea SD = standard deviation; n = frequency; % = percentage

Parameter	n = 200 (%)
Age, mean (SD), years	62 (12)
Hours of night-time use, mean (SD)	7 (1)
Residual apnoea-hypopnoea index, mean (SD)	0.84 (1.9)
Gender	
Male	120 (60)
Female	80 (40)
Level of education	
Primary	72 (36)
Secondary	54 (27)
Upper secondary	23 (11.5)
Bachelor’s degree	28 (14)
Vocational	17 (8.5)
Post-graduate	3 (1.5)
Master’s degree	3 (1.5)
Marital status	
Single	38 (19)
Married	120 (60)
Widower	30 (15)

The mean age and low SOC showed a mean age of 61 years, with a p-value of 0.847; 13% (n = 26) of males had low SOC, with a p-value of 0.087 and a Phi value of 0.121. Regarding educational level and low SOC, 10% (n = 20) reported primary education, with a p-value of 0.34. In terms of marital status and low SOC, 3% (n = 6) reported being single, with a p-value of 0.140 (Table 2).

**Table 2:** Sociodemographic characteristics related to the sense of coherence SOC = sense of coherence; CI = confidence interval; n = frequency; 1 = Student’'s t-test for independent samples; 2 = Pearson's chi-squared test; 3 = linear trend test; * = small effect size: 0.1-0.2

Parameter	Low SOC n = 52 (%)	High SOC n = 148 (%)	p-value	Phi* and Cramér’s V
Age, mean (SD), years	61 (12)	63 (12)	0.847 ^1^	
Gender				
Male	26 (13)	94 (47)	0.087 ^2^	0.121*
Female	26 (13)	54 (27)		
Level of education				
Primary	20 (10)	52 (26)	0.345 ^3^	
Secondary	17 (8.5)	37 (18.5)		
Upper secondary	8 (4)	15 (7.5)		
Bachelor’s degree	5 (2.5)	23 (11.5)		
Vocational	2 (1)	15 (7.5)		
Post-graduate	0 (0)	3 (1.5)		
Master’s degree	0 (0)	3 (1.5)		
Marital status				
Single	6 (3)	32 (16)	0.140 ^3^	
Married	35 (17.5)	85 (42.5)		
Widower	10 (5)	20 (10)		
Cohabiting	1 (0.5)	11 (5.5)		

The mean age and non-adherence to treatment showed a mean age of 64 years, with a p-value of 0.774; 12.5% (n = 25) of female participants were non-adherent, with an OR of 1.14 (95% CI: 0.62-2.13), p = 0.658, and a Phi value of 0.658. With respect to educational level and non-adherence, 11% (n = 22, p = 0.793) reported having completed primary education. Moreover, regarding marital status and non-adherence, 4% (n = 8) stated they were single, with a p-value of 0.376 (Table 3).

**Table 3:** Sociodemographic characteristics associated with CPAP adherence CI = confidence interval; n = frequency; 1 = Student's t-test for independent samples; 2 = Pearson's chi-squared test; 3 = linear trend test; CPAP = continuous positive airway pressure

Parameter	Non-adherence n = 59 (%)	Adherence n = 141 (%)	OR (95% CI)	p-value
Age, mean (SD), years	64 (10)	62 (12)	-	0.774 ^1^
Gender				
Female	25 (12.5)	55 (27.5)	1.14 (0.62-2.13)	0.658 ^2^
Male	34 (17)	86 (43)		
Level of education				
Primary	22 (11)	50 (25)	-	0.793 ^3^
Secondary	19 (9.5)	35 (17.5)		
Upper secondary	5 (2.5)	18 (9)		
Bachelor’s degree	8 (4)	20 (10)		
Vocational	4 (2)	13 (6.5)		
Post-graduate	1 (0.5)	2 (1)		
Master’s degree	0 (0)	3 (1.5)		
Marital status				
Single	8 (4)	30 (15)	-	0.376 ^3^
Married	40 (20)	80 (40)		
Widower	9 (4.5)	21 (10.5)		
Cohabiting	2 (1)	10 (5)		

The association between the residual API and non-adherence to CPAP therapy was 26.5% (n = 53), with an OR of 0.898 (95% CI: 0.824-0.979), p = 0.001, whilst 74% (n = 148) were reported to have a high SOC, with an OR of 0.885 (95% CI: 0.802-0.976), p = 0.000. With regard to hours of night-time use and non-adherence to CPAP therapy, 5% (n = 10) were reported, with an OR of 0.831 (95% CI: 0.740-0.932), p = 0.001, whereas among those with a high SOC, 2.5% (n = 5) were reported, with an OR of 2.846 (95% CI: 0.858-9.437), p = 0.130. In terms of weekly usage of fewer than five days and non-adherence to CPAP, the rate was 22% (n = 44), with an OR of 0.254 (95% CI: 0.164-0.394), p = 0.001, whereas among those with a high SOC, the rate was 5.5% (n = 11), with an OR of 8.538 (95% CI: 4.663-15.633), p = 0.000 (Table 4).

**Table 4:** Sleep trends and patterns related to CPAP adherence and sense of coherence n = frequency; % = percentage; SOC = sense of coherence; CI = confidence interval; OR = odds ratio; 1 = Pearson's chi-squared test; CPAP = continuous positive airway pressure

Parameter	Non-adherencence n = 59 (%)	Adherence n = 141 (%)	OR with 95% CI	p-value	High SOC n = 148 (%)	Low SOC n = 52(%)	OR with 95% CI	p-value
Residual apnoea-hypopnoea index								
Adequate control	53 (26.5)	141 (70.5)	0.898 (0.824-0.979)	0.001 ^1^	148 (74)	46 (23)	0.885 (0.802-0.976)	0.000 ^1^
Mild persistence	6 (3)	0 (0)			0 (0)	6 (3)		
Hours of night-time use								
Less than 5 hours	10 (5)	0 (0)	0.831 (0.740-0.932)	0.001 ^1^	5 (2.5)	5 (2.5)	2,846 (0.858-9.437)	0.130 ^1^
More than 5 hours	49 (24.5)	141 (70.5)			143 (71.5)	47 (23.5)		
Days of use per week								
Fewer than 5 days	44 (22)	0 (0)	0.254 (0.164-0.394)	0.001 ^1^	11 (5.5)	33 (16.5)	8,538 (4.663–15.633)	0.000 ^1^
More than 5 days	15 (7.5)	141 (70.5)			137 (6.5)	19 (9.5)		

Participants with high SOC showed higher levels of CPAP treatment adherence (21%, n = 42) compared with those who did not adhere (2.5%, n = 5). Statistical analysis revealed a significant association between high SOC and treatment adherence (p = 0.00), Cramer's V = 0.473 (Table 5).

**Table 5:** Comparison between levels of sense of coherence and adherence to CPAP treatment SOC = sense of coherence; n = frequency; % = percentage; 1 = chi-squared test for linear trend; * = moderate association 0.3-0.5; CPAP = continuous positive airway pressure

SOC	Adherence n = 141 (%)	Non-adherence n = 59 (%)	Total n = 200 (%)	p-value	chi-squared value	Cramér’s V
High	42 (21)	5 (2.5)	47 (23.5)	0.00^1^	44.8	0.473*
Medium	81 (40.5)	20 (10)	101 (50.5)			
Low	18 (9)	34 (17)	52 (26)			
Total	141 (70.5)	59 (29.5)	200 (100)			

In the multivariate model for CPAP non-adherence, low SOC was associated with an OR of 9.043 (95% CI: 4.359-18.761). Single marital status was associated with an OR of 1.434 (95% CI: 0.685-3.001). With regard to age over 65 years, the OR was 0.745 (95% CI: 0.200-2.767). The male gender had an OR of 1.111 (95% CI: 0.532-2.322) (Table 6).

**Table 6:** Multivariate model: factors associated with CPAP non-adherence OR = odds ratio; CI = confidence interval; R² = coefficient of determination; P = overall model percentage; Ref. = reference category; bootstrapping = 3,000; CPAP = continuous positive airway pressure

Logistic regression with bootstrapping	Parameter	Beta	Wald test	OR with 95% CI using bootstrapping	p-value
Nagelkerke R² = 0.274, Hosmer-Lemeshow test = 2.965, p=0.888, P = 78.5%	Constant	-0.668	1.006	-	0.513
	Low SOC (Ref.: High SOC)	2.202	34,974	9.043 (4.359-18.761)	0.005
	Marital status: single (Ref.: married)	0.36	0.913	1,434 (0.685-3.001)	0.339
	Advanced educational level (Ref.: basic educational level)	-0.321	0.641	0.726 (0.331-1.591)	0.423
	Age > 65 years old (Ref.: < 65 years old)	-0.295	0.194	0.745 (0.200-2.767)	0.66
	Male (Ref.: female)	0.105	0.078	1.111 (0.532-2.322)	0.779

## Discussion

This study analysed the association between low SOC and non-adherence to CPAP therapy in a sample of 200 patients with OSA [11]. This can be explained on the basis of empirical evidence suggesting that treatment adherence is primarily influenced by behavioural and cognitive factors, such as the perception of the severity of the condition, initial experience with the device, self-efficacy, and social support [12].

A key finding was the association between SOC and treatment adherence. The results showed a statistically significant relationship between SOC and CPAP adherence rates, with a higher percentage of adherence observed in patients with high SOC and a higher rate of non-adherence in patients with low SOC. These findings suggest that SOC acts as a protective psychological resource that contributes to adaptation to treatment and therapeutic persistence. From a salutogenic perspective, these individuals tend to perceive the demands of the condition as understandable and manageable, which could facilitate tolerance of the initial discomfort associated with CPAP, considered one of the main barriers during the first few weeks of treatment [13].

The literature supports these findings; it has been reported that adherence depends largely on psychosocial factors, particularly the patient's perception of benefit, motivation, and coping ability [14]. The SEMSA study demonstrated that self-efficacy and positive beliefs about treatment are better predictors of adherence than some traditional demographic factors [12,15]. Similarly, recent research has identified that the perception of early clinical improvement and ongoing medical support significantly increase sustained use of CPAP [14,16]. The results also showed that patients who adhered to treatment had better control of residual AHI, used the device for more than five hours per night more frequently, and used the device on more than five days per week. These findings reinforce the importance of adherence to treatment in achieving adequate clinical control of the condition.

Although no significant differences were observed between high and low SOC in terms of hours of nightly use, there were significant differences in weekly frequency of use and control of residual AHI, suggesting that SOC may have primarily influenced the consistency of treatment rather than the specific duration of nightly use. These results are consistent with recent research indicating that use exceeding four to five hours per night is associated with a reduction in residual AHI, less daytime sleepiness, and better cardiovascular and metabolic outcomes [16,17]. Furthermore, it has been shown that the most effective non-pharmacological interventions for improving adherence are those focused on behavioural support, therapeutic education and motivational strategies [18,19]. The MEntA programme demonstrated significant increases in hours of CPAP use and in the perception of personal competence, highlighting the importance of motivation and self-efficacy in treatment adherence [20]. These dimensions are closely related, in conceptual terms, to the manageability and meaningfulness components of the SOC [5].

Moreover, this study indicates a clinically relevant moderate association between SOC and treatment adherence; although this is a moderate association, its clinical relevance is significant given the multifactorial nature of CPAP adherence, which is typically influenced simultaneously by disease severity, family support, perception of symptoms, anxiety, depression, and professional support [21].

The median CPAP usage of seven hours observed in this sample exceeds the internationally recommended minimum adherence threshold (>4 hours per night), suggesting adequate overall adherence, subject to accounting for baseline AHI due to underlying pulmonary pathology and its severity [22]. However, the persistence of a significant proportion of non-adherent patients supports the existence of a vulnerable group in which interventions aimed at strengthening SOC could have a significant therapeutic effect.

In the multivariate model, other variables, such as gender, age, marital status, and educational level, did not show a significant association. These results are consistent with research highlighting the greater relevance of psychological factors over traditional sociodemographic determinants in the therapeutic behaviour of patients with OSA [21,22].

Strengths

An appropriate multivariate model was used to estimate risks and address the multiple causes; bootstrapping (resampling) was performed, which provided greater confidence in the results obtained with a sample size of up to 2,000, and these results were further validated to show that high SOC was the sole independent predictor of CPAP adherence.

Overall, the results of this study provide relevant evidence positioning the SOC as a constructive theoretical and clinical tool and support a biopsychosocial approach to the management of OSA, the assessment of which could be incorporated into the comprehensive evaluation of individuals with OSA to identify those at greater risk of discontinuation and to develop strategies aimed at strengthening coping and self-care resources.

Limitations

Finally, although the findings suggest an association between SOC and treatment adherence, the cross-sectional design of the study limits the ability to establish definitive causal relationships. Therefore, future longitudinal studies will clarify the possible bidirectional relationships between the two variables and determine whether strengthening SOC through specific interventions can lead to long-term improvements in CPAP treatment adherence [18].

A recognised methodological limitation is that the treatment adherence variable was generated solely for the statistical purposes of this study. Nevertheless, the use of validated and appropriate instruments that allow for an objective and standardised assessment of CPAP use in clinical practice is essential.

Despite the results obtained, this study has some limitations. The use of self-administered questionnaires may contribute to memory bias or social desirability bias.

A major limitation of this study is that it was conducted in a single medical unit, which may make it difficult to extrapolate the results to other populations or care settings. A multicentre study is proposed as a follow-up.

It is recognized that the use of a non-probability convenience sampling method and a simple imputation method may lead to information bias. Furthermore, the absence of the variable comorbidities restricts the external validity of the findings.

## Conclusions

The results of this study suggest that SOC is significantly associated with adherence to CPAP therapy in individuals with OSA. Taken together, the results of this study provide relevant evidence positioning the SOC as a constructive theoretical and clinical tool and support a biopsychosocial approach to the management of OSA, the assessment of which could be incorporated into the comprehensive evaluation of patients with OSA to identify those at greater risk of treatment discontinuation and to develop strategies aimed at strengthening coping and self-care resources.
